# Comparison of the Data Classification Approaches to Diagnose Spinal Cord Injury

**DOI:** 10.1155/2012/803980

**Published:** 2012-03-05

**Authors:** Yunus Ziya Arslan, Rustu Murat Demirer, Deniz Palamar, Mukden Ugur, Safak Sahir Karamehmetoglu

**Affiliations:** ^1^Department of Mechanical Engineering, Faculty of Engineering, Istanbul University, Avcilar, 34320 Istanbul, Turkey; ^2^Department of Mathematics and Computer Science, Istanbul Kultur University, Sirinevler, 34156 Istanbul, Turkey; ^3^Department of Physical Medicine and Rehabilitation, Kars State Hospital, 36000 Kars, Turkey; ^4^Department of Electrical & Electronics Engineering, Faculty of Engineering, Istanbul University, Avcilar, 34320 Istanbul, Turkey; ^5^Department of Physical Medicine and Rehabilitation, Cerrahpasa Medical Faculty, Istanbul University, Cerrahpasa, 34098 Istanbul, Turkey

## Abstract

In our previous study, we have demonstrated that analyzing the skin impedances measured along the key points of the dermatomes might be a useful supplementary technique to enhance the diagnosis of spinal cord injury (SCI), especially for unconscious and noncooperative patients. Initially, in order to distinguish between the skin impedances of control group and patients, artificial neural networks (ANNs) were used as the main data classification approach. However, in the present study, we have proposed two more data classification approaches, that is, support vector machine (SVM) and hierarchical cluster tree analysis (HCTA), which improved the classification rate and also the overall performance. A comparison of the performance of these three methods in classifying traumatic SCI patients and controls was presented. The classification results indicated that dendrogram analysis based on HCTA algorithm and SVM achieved higher recognition accuracies compared to ANN. HCTA and SVM algorithms improved the classification rate and also the overall performance of SCI diagnosis.

## 1. Introduction

The diagnosis of spinal cord injury (SCI) by neurological examination technique depends mainly on the experience of medical doctor and hence may lead to nonobjective ways of assessment [1]. In this technique, doctors assess the patient's symptoms, which may comprise of loss of motor or sensory function. McDonald and Sadowsky [2] reported that assessment should include mental status, cranial nerves, motor, sensory and autonomic systems, coordination, and gait. Patient symptoms may include extreme pain or pressure in the neck, head, or back; loss of sensation in the hand, fingers, feet, or toes; partial or complete loss of control over any part of the body and much more. Since this traditional technique requires continuous feedback, it has some limitations especially for noncooperative and unconscious patients. For clinical applications, such as monitoring the treatment and rehabilitation processes following a surgery, a more quantitative and objective way for the diagnosis of spinal cord injury is required. In recent years, some encouraging investigations have been carried out for this purpose by assessing the thermal [3] and electrical perception threshold [4, 5]. However, these techniques require patient's feedback, hence cannot be effectively used for noncooperative and unconscious patients. Roehl et al. [6] examined the temperature difference on the skin surface by evaluating the thermographic imaging, and they concluded that thermography could be prospectively used as a supplement to existing diagnostic measures for SCI.

Recently we have suggested a new method, which can eliminate the patient-feedback dependency for the diagnosis of SCI in a quantitative manner [7]. This technique, which was based on the artificial neural networks (ANN) [8–10], could distinguish between skin impedances of control subjects and patients satisfactorily. In the present study, by using alternative algorithms, it was aimed to improve the diagnosing performance of the proposed method. To achieve this goal, in addition to ANN, support vector machine (SVM) and dendrogram-based hierarchical cluster tree analysis (HCTA) approaches were also used as alternative methods for the classification of the impedance values.

SVM is a supervised machine-learning algorithm based on a statistical learning theory approach for solving data classification and pattern recognition problems [11]. Although the fundamental concept of SVM was established in the late seventies [12], this method began to be widely used in the mid of nineties (for review, see [13]). In biomedical applications, this technique is frequently employed [14–16].

Dendrogram-based cluster analysis was first appeared in the study of Sneath and Sokal [17]. In this analysis method, data (objects) are divided into groups (clusters) that share common characteristics [18]. HCTA is one of the types of the cluster analysis, which is basically based on calculating the distances between data and finally grouping them into a hierarchical cluster tree (dendrogram) according to these distances. In biology, clustering analysis has been especially used to find groups of genes that have similar functions [18].

## 2. Methods

### 2.1. Experimental Procedure

The impedance data analyzed in this study were previously collected for another reported study, hence a detailed explanation of the experimental protocol can be found in [7]. However, for the sake of completeness, a condensed form of the experimental procedure was given below. 

#### 2.1.1. Subjects

Patients with traumatic SCI and control subjects aged between 18 and 55 years were included in the study. Duration of injury of the patients varied from three to twenty years. Initially, they were all evaluated by history and physical examination according to The International Standards for Neurological Classification of SCI, American Spinal Injury Association (ASIA), and International Spinal Cord Society (ISCoS) [1]. All procedures were approved by the Ethical Committee of Cerrahpasa Medical Faculty, Istanbul University.

**Figure 1:**
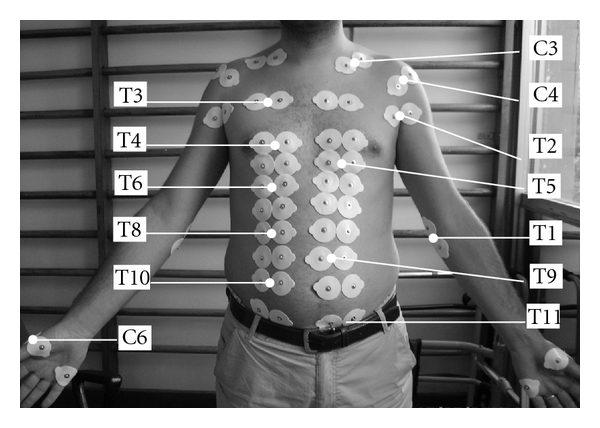
Location of the some of the sensory key points.

Skin impedances of the key points between C3 and S1 were measured in 15 control subjects and 15 patients with SCI (13 paraplegics and 2 tetraplegics) bilaterally (Figure 1). The impedances were measured in all dermatomes except C2 (due to hair), L1-3, and S2-5 (because of the refusal of the control subjects). According to the aforementioned booklet of ASIA and ISCoS, 10 pairs of key muscles and 28 pairs of key points were evaluated and finally the neurological level, completeness, and classification of SCI were determined. For the patients, inclusion criteria were determined as traumatic SCI and both gender; however, the exclusion criteria were determined for patients with any other neurological disorder than SCI and also nontraumatic SCI.

#### 2.1.2. Skin Impedance Measurement

**Figure 2:**
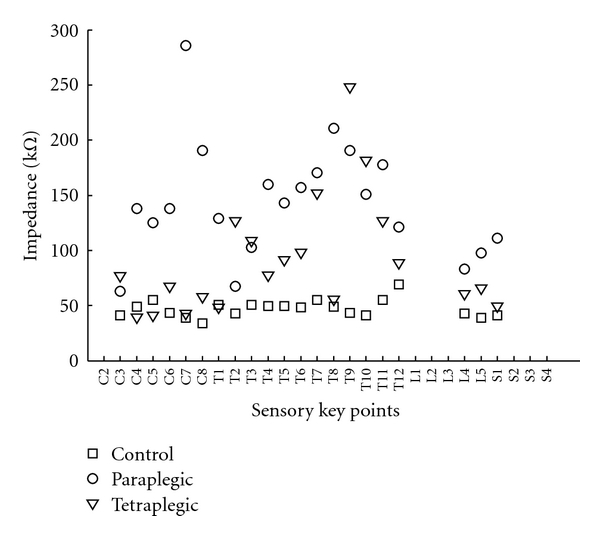
Impedance data obtained from representative control, paraplegic, and tetraplegic subjects.

In order to simulate the worst case condition, skin was not prepared artificially by abrasion or cleaning with alcohol before the measurements. Two self-adhesive electrodes were placed on the skin for each key point, and an AC signal (2 V, 200 Hz) was applied by means of a signal generator. The electrodes were placed on either side of the sagittal plane of the body. A portable multimeter was situated between one of the electrodes and signal generator, and the current level was recorded. The other output of the signal generator was connected to the electrode, which was not fixed to the multimeter. All experiments were performed by using electrocardiography (ECG) type electrodes (Unomedical, Unilect). The distance between the centers of the electrodes was 3 cm. In order to prevent the deterioration of adhesiveness of electrodes, which can eventually affect the skin-electrode impedance, each electrode was used once. In Figure 2, representative data obtained from control, paraplegic and tetraplegic subjects can be seen.

### 2.2. Artificial Neural Networks

Neural Networks are mathematical models inspired by the human brain. These models consist of processing layers, where each node in a given input, hidden or output layers represent a neuron of that layer. They possess ability to approximate any arbitrary input-output mapping function, by learning like backpropagation and adapting parameters to training data and ability to generalize new testing data even from a lack of statistical knowledge about the input data [9]. Learning process of the ANN occurs at the synaptic junctions between the neurons of the input layer and the neurons of the output layer [8].

Dimension of the structure of an ANN considerably affects its classification performance. It is well accepted that networks with large dimensions (large number of hidden layers and neurons) do not always improve the accuracy of the classification process [19]. Moreover, neural networks with large dimensions do not converge easily and may be very time-consuming during the training process. However, small networks may fall into a local error minimum and subsequently learning from training data may not be optimal. In this study, since a three layer network (two hidden layers) can approximate any nonlinear function [20], we used two hidden layers in the network model. We determined the numbers of neurons per layer by grid search. In the network structure, one input layer, two hidden layers, and one output layer have 27, 16, 6, and 1 neurons, respectively. The input array was constituted from the mean values of skin impedances of the left-and right-side key points according to sagittal plane and target array was constituted from array of ones (denotes patients) and zeros (denotes subjects).

Transfer function is used mainly for the calculation of weight factor between neurons during the training process. In our case, we used log-sigmoid transfer function, since its output range (0 to 1) is ideal to output Boolean values. Backpropagation feed-forward algorithm was chosen for the training process, because it has been proven to be a robust algorithm for difficult connectionist learning problems [21]. Backpropagation algorithm is an extension of the least mean square learning algorithm and is widely used in adaptive signal processing. The weights are adjusted at each step to reduce the gradient of the cost function [8, 9]. Number of the epoch was limited to 500 for the learning stage of the network.

We built a matrix of training including skin impedance values measured from control and patient subjects. Each row corresponds to a measured skin impedance values, and each column corresponds to a subject. Once we trained the ANN, we classified test subjects including both patient and control subjects disjointed from training set. We then measured performance of ANN on the test subjects. During the training and testing phase, we implemented 10-fold cross-validation technique which is based on shuffling sample vectors among training and testing space randomly [22]. This method aims to maximize the amount of data that can be used for training to ensure a model that will generalize well to unseen data. In this technique, the impedance data set (30 subjects) was divided into 10 subsets; each subset consisted of three subjects. Training of the ANN was repeated 10 times. Each time, a single subset was retained as the validation impedance data for testing, and the remaining nine subsets (27 subjects) were used as training data. After cross-validation was completed for all of the subjects, means of the 10 classification results were computed.

### 2.3. Support Vector Machines

This method is a kernel-based classification technique that is based on the margin-maximization principle that minimizes an upper bound on the expected loss (risk) using observed data [23, 24]. In this method, the goal is to estimate the influence of an input measurement $X\in \{{\vec{x}}_{1},{\vec{x}}_{2},\ldots ,{\vec{x}}_{n}\}$ variable on an output classification variable *Y* ∈ {*y*_1_, *y*_2_,…, *y*_*n*_} to find an optimal predictor *f* : *X* → *Y*, that is, a kernel function. In our case, *n* was defined as 30, since we have 30 subjects.

The SVM algorithm finds the decision boundary function as a linear combination of high-dimensional support vectors, which are acquired from training pair of examples from a sample space $S_{n}\;=\;\left({\vec{x}}_{1},y_{1}\right),\ldots ,\left({\vec{x}}_{n},y_{n^{\prime }}\right)\in X\times Y$, (independent and identically distributed) values from an unknown probability distribution, where *n*′ = 27 which corresponds to training sample size. This value denotes size of subset of all cases satisfying (*n*′ < *n* = 30) for training set. Each vector comprised of 21 dimensional skin impedance values corresponding to a patient or healthy subject.

The hyperplane can classify two classes in SVM machines when we set kernel function and regularizing parameter *C* appropriately. If dist^+^ and dist^−^ are becoming the shortest distances to this separating hyperplane bordering two classes, then the margin of the separating hyperplane becomes |dist^+^ − dist^−^|. The shortest distance and the normal direction (orthogonal) to the hyperplane are related to each other. $\vec{w}$ is the 21-dimensional weight vector which is a function of the distance, $\text{dis}{\text{t}}^{+}=\text{dis}{\text{t}}^{-}=1/||\vec{w}||$. Maximizing the margin means minimizing the term ||*w*||/2, which shows the best classification success between patients and healthy subjects.

Every training example $z=({\vec{x}}_{i},y_{i})$ consisted of a vector including the impedance values of key points *x*_*i*_ ∈ *ℜ* ^21^, and a discrete classification label value (binary classification), which corresponded to two groups (patients (−1) and controls (1)). Our goal was to predict the label value ${\hat{y}}_{i}\in \{-1,1\}$ using other test set which included a mixture of vectors with two states. In the training stage, we had previously done searching for an optimal hyperplane, which maximizes margin and minimizes errors with known corresponding labels *y* ∈ {−1,1} included in the training set. In testing phase, kernel function led to predict the patients and control subjects.

The effectiveness of SVM depends mainly on the selection of the kernel, the kernel parameters, and regularization parameter *C*. In our case, we selected the radial basis function kernel (Gaussian kernel), because it is very flexible and can adapt in complexity to fit the training data. The Gaussian kernel parameter, *σ*, determines the area of influence of the support vector over the data space. Regularization parameter, *C*, controls the tradeoff between margin maximization and error minimization. In order to find the optimal values for the kernel parameter and regularization parameter, we used cross-validation and grid search. After grid search, we obtained *σ* as 0.1 and *C* as 10.

To be able to compare the performances of ANN and SVM in equivalent conditions, 10-fold cross-validation technique used for ANN was also employed for SVM.

### 2.4. Dendrogram Analysis Based on Hierarchical Cluster Tree Analysis

In our specific case, hierarchical cluster tree analysis is a way to create groups of subjects in such a way that the skin impedance values of subjects in the same cluster are very close in magnitudes, and the skin impedance values of subjects in different clusters are quite different. Hierarchical clustering analysis divides whole tree into lower branches (leaves) as necessary.

In our study, we established a hierarchical evaluation structure [25] in a tree *T*, which can be considered as a clustering process for grouping different objects together. The root of the dendrogram denotes the entire data set including control and patient groups. This hierarchical tree consists of many U-shaped lines connecting patients and control subjects. The height of each U represents the distance between the two objects being connected. This method builds up a hierarchical classification in a bottom-up way from leaves up to the roots of the tree ordering with a distance matrix *D*. The distance matrix contains dissimilarity values among pair of individuals (patients and control subjects) $\Omega =\{{\vec{x}}_{1},{\vec{x}}_{2},\ldots ,{\vec{x}}_{n}\}\in T$.

In the first step, since we initially did not know which individual belongs to either patient or control subject class, we initialized all 15 patients and 15 control subjects with singleton clusters (sets with exactly one element) which means that we had a total of 30 clusters with each cluster containing just single patient or control subject as for all $\vec{x}\in \Omega$, $\{\vec{x}\}\in T$ at the very beginning. In other words, we formed subtrees $\left\{{\vec{x}}_{1}\},\{{\vec{x}}_{2}\},\ldots ,\{{\vec{x}}_{30}\right\}$. Then, we computed the Euclidian distances $D(\{{\vec{x}}_{i}\},\{{\vec{x}}_{j}\})=\sqrt{({\vec{x}}_{i}-{\vec{x}}_{j}){\left({\vec{x}}_{i}-{\vec{x}}_{j}\right)}^{T}}$ (∀*i*, *j* = 1,2,…, 30 and *i* ≠ *j*) between those singleton clusters. Once the proximity between subjects has been computed, we linked pairs of subjects that are close together into clusters made up of two subjects (binary clusters). We then linked these newly formed subjects to each other and to other subjects to create bigger clusters until all the subjects in the original data set are linked together in a hierarchical tree. Since it was aimed to observe the natural divisions that exist among links between subjects, we did not apply a clustering threshold.

## 3. Results

Since the ANN and SVM methods require cross-validation analysis, statistical significance analysis was only performed between the classification results of ANN and SVM (in our case, “classification result” refers to as percent of cases in which the different computational algorithms correctly predict whether or not an individual has a SCI). One-way ANOVA was used to analyze the statistically significant difference between means of the classification results of ANN and SVM. However, HCTA does not require training and cross-validation processes, that is, data set is evaluated as a whole rather than divided into subsets for training and testing phases. Therefore, there is no need to calculate an average of the validation result. For this reason, it cannot be performed a statistical significance analysis for HCTA. The level of significance was preset for all statistics at *P* = 0.05.

**Figure 3:**
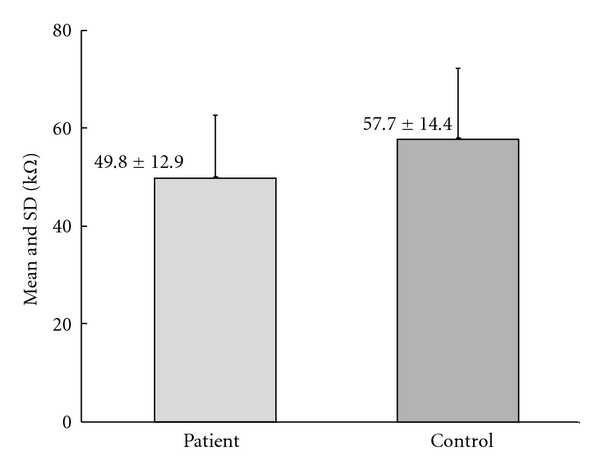
Mean and standard deviation (SD) of the magnitudes of the skin impedance values of all subjects.

Mean and standard deviation (SD) of the magnitudes of the skin impedances of all subjects (controls and patients) are denoted in Figure 3. No statistically significant difference between mean impedance values of controls and patients was found.

Since the number of the tetraplegics (only two patients; due to the inconvenient and difficult situations in measuring the skin impedances of tetraplegics) was much smaller than that of the paraplegics in the patient group, two different data sets were utilized during the data classification process. In doing so, it was intended to observe the effect of insufficient number of tetraplegics on the classifying results. The classification process, in which all the subjects were included, is referred to as Phase I (control + paraplegics + tetraplegics) and the other process, in which only control and paraplegic groups were included, is referred to as Phase II (control + paraplegics).

The average success rate of the classification results of the ANN and SVM was obtained as 73.3% and 78.5% for Phase I, respectively (Table 1). For Phase II, means of the classification results of the ANN and SVM were obtained as a rate of 76.6% and 100%, respectively. In addition, classification results of HCTA were 83.3% and 85.7% for Phase I and Phase II, respectively.

**Figure 4:**
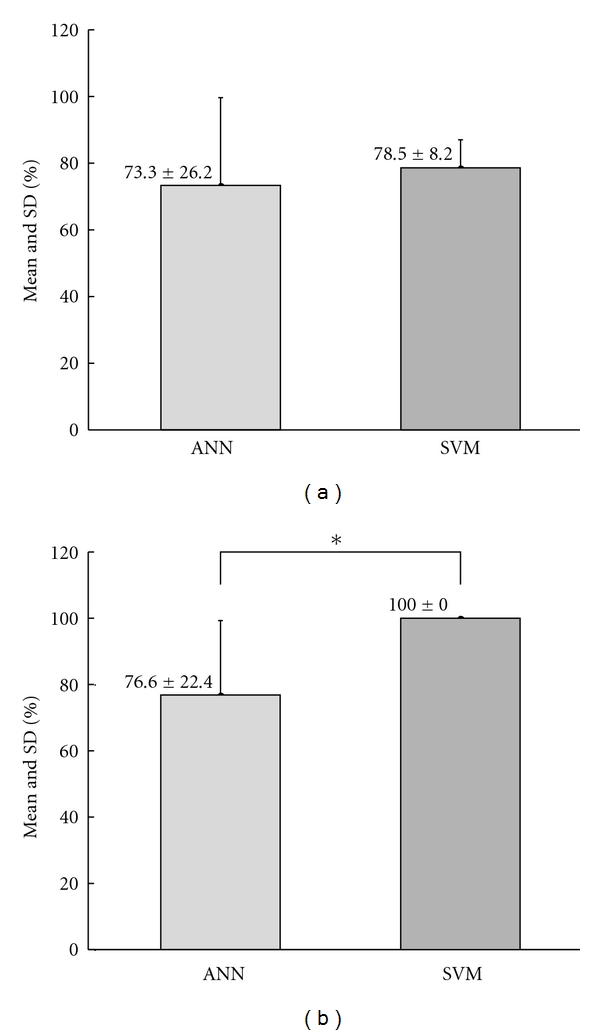
Mean and standard deviation (SD) of the classification results of ANN and SVM for (a) Phase I (paraplegic + tetraplegic + control) and (b) Phase II (paraplegic + control) (*P < 0.05).

A comparison of the classification performances of ANN and SVM in diagnosing SCI is presented in Figure 4. In Phase I (Figure 4(a)) and Phase II (Figure 4(b)), means of the validation results obtained by SVM are higher than those obtained by ANN. A statistically significant difference between validation results of ANN and SVM was found for Phase II, but not for Phase I.

**Figure 5:**
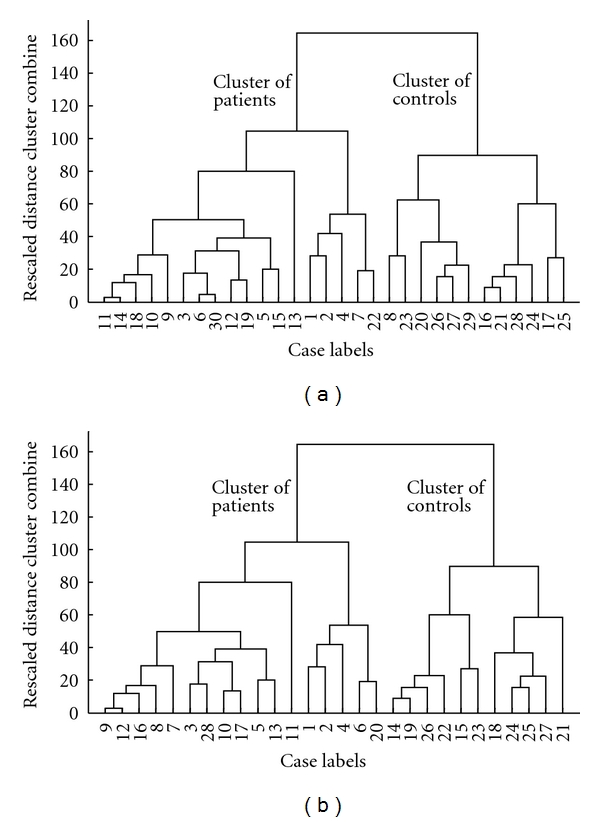
Dendrogram diagrams indicating the relationship between patients with SCI and control subjects (a) Phase I. Patients with SCI (paraplegics + tetraplegics) are denoted by 1–15 and control subjects are denoted by 16–30. (b) Phase II. Patients with SCI (only paraplegics) are denoted by 1–13 and control subjects are denoted by 14–28.

The dendrogram can be described as a graphical representation of the results of hierarchical cluster analysis. Dendrograms of the HCTA are given for Phase I and Phase II in Figure 5. In this figure, numbers along the horizontal axis and along the vertical axis represent the indices of the subjects (patients and controls) in the original data set and Euclidean distance between the skin impedances of the connected subjects, respectively. In Figure 5(a), patients with SCI (paraplegics + tetraplegics) and control subjects are denoted by the numbers 1 to 15 and 16 to 30, respectively. In Figure 5(b), patients with SCI (only paraplegics) are denoted by the numbers 1–13, and control subjects are denoted by the numbers 14 to 28.

As shown in Figure 5(a), for Phase I, 25 out of 30 subjects fell in the correct clusters (83.3%), whereas 5 out of 30 subjects (viz., 18, 30, 19, 22, and 8) fell in the wrong cluster. As shown in Figure 5(b), for Phase II, 24 out of 28 subjects fell in the correct cluster (85.7%), whereas 4 out of 28 subjects (viz., 16, 28, 17, and 20) fell in the wrong cluster.

In order to allow visualization of the classification performances of the three algorithms, confusion matrices were given in Tables 2(a)–2(f). Each column of the matrices represents the instances in the predicted class, while each row represents the instances in the actual class. All correct classifications are located in the diagonal of the tables.

## 4. Discussion

In our case, hierarchical clustering analysis utilized all available patient and control data in two steps. Initially, it calculated the distance between pair of subjects, which were selected and grouped together according to their impedance values. Later on, similar groups were selected and joined together, which led new and bigger groups. This process continued until all subjects were selected and attached to part of the tree. In our case, the tree had two major branches, which were formed by patients and healthy subjects. This new approach extracted patients and healthy subjects satisfactorily from an unlabeled set without invoking patient feedback. Dendrogram analysis does not require cross-validation; hence, it is computationally efficient.

ANN and SVM require some design parameters which are actually not known a priori. In case of SVM, if the regularization parameter is not selected properly, it might cause overfitting or underfitting. In case of ANN, dimension of network structure, initial weights, number of iterations, transfer function, and learning rate affect the accuracy of the classification considerably [19]. There have been numerous studies on the determination of the optimum neural network structure; however, a consensus on a certain approach to determine the best structure has not been reached [26]. These parameters could be estimated from cross-validation technique; however, it needs extra time and risk. In contrast to ANN, SVM algorithm automatically selects its model size [27]. Moreover, SVM training always finds a global minimum, whilst ANN optimization is often susceptible to local minima [13]. In addition to these advantages of SVM over neural networks, Shawe-Taylor and Cristianini [28] indicated more key features of SVM, such as the use of kernels, the sparseness of the solution, and the capacity control obtained by optimizing the margin.

Validation results obtained by using SVM were in agreement with those obtained by ANN. In both cases, rates of diagnosis of SCI with success were higher for Phase II than those for Phase I. The reason for this difference in the validation rates of Phase I and Phase II stems from the lack of the sufficient number of tetraplegic subjects. The performance of the algorithms used in classifying the patients with SCI and controls depends considerably on the number of subjects used as input in the training stage. ANN showed a modest increase in percentage accuracy from Phase I to Phase II. On the other hand, SVM showed a much larger increase in accuracy when the tetraplegic patients are absent because a multilayer neural network classifier suffers from the existence of multiple local minima solutions, whereas SVM is formulated as a quadratic programming problem and hence SVM training always finds a global minimum [13].

The results showed that HCTA and SVM algorithms improved the classification rate and also the overall performance. For Phase I, hierarchical clustering analysis achieved higher recognition accuracy compared to ANN and SVM systems; however, for Phase II, SVM showed the best classification performance.

Since the neurological examination technique used in the diagnosis of the SCI is mostly subjective, an objective and accurate technique would be a very important improvement for clinical applications. The suggested quantitative method in which the skin impedances were classified using the hierarchical clustering or SVM is a quite simple, noninvasive, and nonexpensive method. A multimeter, a frequency generator, ECG electrodes and a computer are sufficient to perform this technique. Moreover, measurement and analysis of the impedance do not require patient feedback, which ensures this technique to be applicable as a more objective method, especially for unconscious and noncooperative SCI patients.

It is concluded that the proposed skin impedance test based on SVM or HCTA can be used as a supplement to neurological and radiological examinations to enhance the diagnosis of SCI. For future studies, measurements of skin impedance of acute patients are planned. Also, other distinctive parameters, such as skin temperature [6], for diagnosing patients with SCI injury among healthy people, can be taken into account together with skin impedance. Such a combination of these distinctive parameters might improve the accuracy of the diagnosis of SCI.

## Figures and Tables

**Table 1:** Classification results of the patients and control subjects obtained using ANN, SVM, and HCTA.

	ANN	SVM	HCTA
Phase I (paraplegic + tetraplegic + control)	73.3%	78.5%	83.3%
Phase II (paraplegic + control)	76.6%	100%	85.7%

Results of the ANN and SVM approaches shown in this table are the mean values obtained from 10-fold cross-validation. Statistically significant difference between means of the classification results of ANN and SVM was found only for Phase II.

**Table:** (a)

		Predicted class	
		Control subject	Patient subject

Actual class	Control subject	11	4
	Patient subject	4	11

**Table:** (b)

		Predicted class	
		Control subject	Patient subject

Actual class	Control subject	11	4
	Patient subject	3	10

**Table:** (c)

		Predicted class	
		Control subject	Patient subject

Actual class	Control subject	12	3
	Patient subject	4	11

**Table:** (d)

		Predicted class	
		Control subject	Patient subject

Actual class	Control subject	15	0
	Patient subject	0	13

**Table:** (e)

		Predicted class	
		Control subject	Patient subject

Actual class	Control subject	11	4
	Patient subject	1	14

**Table:** (f)

		Predicted class	
		Control subject	Patient subject

Actual class	Control subject	11	4
	Patient subject	0	13

## References

[1] Maynard FM, Bracken MB, Creasey G (1997). International standards for neurological and functional classification of spinal cord injury. *Spinal Cord*.

[2] McDonald JW, Sadowsky C (2002). Spinal-cord injury. *The Lancet*.

[3] Nicotra A, Ellaway PH (2006). Thermal perception thresholds: assessing the level of human spinal cord injury. *Spinal Cord*.

[4] Savic G, Bergström EMK, Frankel HL, Jamous MA, Ellaway PH, Davey NJ (2006). Perceptual threshold to cutaneous electrical stimulation in patients with spinal cord injury. *Spinal Cord*.

[5] Savic G, Bergström EMK, Davey NJ (2007). Quantitative sensory tests (perceptual thresholds) in patients with spinal cord injury. *Journal of Rehabilitation Research and Development*.

[6] Roehl K, Becker S, Fuhrmeister C, Teuscher N, Füting M, Heilmann A (2009). New, non-invasive thermographic examination of body surface temperature on tetraplegic and paraplegic patients, as a supplement to existing diagnostic measures. *Spinal Cord*.

[7] Karamehmetoglu SS, Ugur M, Arslan YZ, Palamar D (2009). A quantitative skin impedance test to diagnose spinal cord injury. *European Spine Journal*.

[8] Arslan YZ, Adli MA, Akan A, Baslo MB (2010). Prediction of externally applied forces to human hands using frequency content of surface EMG signals. *Computer Methods and Programs in Biomedicine*.

[9] Haykin S (2008). *Neural Networks: A Comprehensive Foundation*.

[10] Lisboa PJG (2002). A review of evidence of health benefit from artificial neural networks in medical intervention. *Neural Networks*.

[11] Vapnik V (1998). *Statistical Learning Theory*.

[12] Vapnik V (1982). *Estimation of Dependences Based on Empirical Data*.

[13] Burges CJC (1998). A tutorial on support vector machines for pattern recognition. *Data Mining and Knowledge Discovery*.

[14] El-Naqa I, Yang Y, Wernick MN, Galatsanos NP, Nishikawa RM (2002). A support vector machine approach for detection of microcalcifications. *IEEE Transactions on Medical Imaging*.

[15] Noble WS (2006). What is a support vector machine?. *Nature Biotechnology*.

[16] Chen HL, Yang B, Wang G, Liu J, Chen YD, Liu DY A three-stage expert system based on support vector machines for thyroid disease diagnosis.

[17] Sneath PHA PHA, Sokal RR (1973). *Numerical Taxonomy—The Principles and Practice of Numerical Classification*.

[18] Tan PN, Steinbach M, Kumar V (2006). *Introduction to Data Mining*.

[19] Kavzoglu T Determining optimum structure for artificial neural networks.

[20] Nguyen D, Widrow B Improving the learning speed of 2-layer neural networks by choosing initial values of the adaptive weights.

[21] Hecht-Nielsen R Theory of the backpropagation neural network.

[22] Kohavi R A study of cross-validation and bootstrap for accuracy estimation and model selection.

[23] Cortes C, Vapnik V (1995). Support-vector networks. *Machine Learning*.

[24] Schölkopf B, Mika S, Burges CJC (1999). Input space versus feature space in kernel-based methods. *IEEE Transactions on Neural Networks*.

[25] Fernández A, Gómez S (2008). Solving non-uniqueness in agglomerative hierarchical clustering using multidendrograms. *Journal of Classification*.

[26] Wilamowski BM (2009). Neural network architectures and learning algorithms. *IEEE Industrial Electronics Magazine*.

[27] Rychetsky M (2001). *Algorithms and Architectures for Machine Learning Based on Regularized Neural Networks and Support Vector Approaches*.

[28] Shawe-Taylor J, Cristianini N (2004). *Kernel Methods for Pattern Analysis*.

